# Impact of BRCA1 and BRCA2 mutations on ovarian reserve and fertility preservation outcomes in young women with breast cancer

**DOI:** 10.1007/s10815-019-01658-9

**Published:** 2019-12-24

**Authors:** Eleonora Porcu, Giulia Maria Cillo, Linda Cipriani, Federica Sacilotto, Leonardo Notarangelo, Giuseppe Damiano, Maria Dirodi, Ilaria Roncarati

**Affiliations:** Infertility and IVF Unit, University of Bologna, Sant’Orsola – Malpighi University Hospital, Via Massarenti 13, 40138 University of Bologna, Bologna, Italy

**Keywords:** BRCA, Fertility preservation, Breast cancer, Oocyte cryopreservation

## Abstract

**Purpose:**

To determine the impact of BRCA1 and BRCA2 mutations on ovarian reserve and fertility preservation outcome. The main purpose and research question of the study is to determine the impact of BRCA1 and BRCA2 mutations on ovarian reserve and fertility preservation outcomes.

**Methods:**

Prospective study: 67 breast cancer patients between 18 and 40 years old, undergoing a fertility preservation by means of oocyte storage were considered. Inclusions criteria for the study were age between 18 and 40 years old, BMI between 18 and 28, breast cancer neoplasm stage I and II according to American Joint Committee on Cancer classification (2017) and no metastasis. Exclusion criteria: age over 40 years old, BMI < 18 and > 28, breast cancer neoplasm stage III and IV and do not performed the BRCA test. A total of 21 patients had not performed the test and were excluded. Patients were divided into four groups: Group A was composed by 11 breast cancer patients with BRCA 1 mutations, Group B was composed by 11 breast cancer patients with BRCA 2 mutations, Group C was composed by 24 women with breast cancer without BRCA mutations, and Group D (control) was composed by 181 normal women.

**Results:**

Group A showed significant lower AMH levels compared to Group C and D (1.2 ± 1.1 vs 4.5 ± 4.1 *p* < 0.05 and 1.2 ± 1.1 vs 3.8 ± 2.5 *p* < 0.05). BRCA1 mutated patients showed a significant lower rate of mature oocytes (MII) compared to Group C (3.1 ± 2.3 vs 7.2 ± 4.4 *p* < 0,05) and Group D (3.1 ± 2.3 vs 7.3 ± 3.4; *p* < 0,05). Breast cancer patients needed a higher dose of gonadotropins compared to controls (Group A 2206 ± 1392 Group B2047.5 ± 829.9 Group C 2106 ± 1336 Group D 1597 ± 709 *p* < 0,05). No significant differences were found among the groups considering basal FSH levels, duration of stimulation, number of developed follicles, and number of total retrieved oocytes. Regarding BRCA2 mutation, no effect on fertility was shown in this study.

**Conclusions:**

The study showed that BRCA1 patients had a higher risk of premature ovarian insufficiency (POI) confirmed by a diminished ovarian reserve and a lower number of mature oocytes suitable for cryopreservation.

## Introduction

Recent studies suggest the possibility that BRCA1 and BRCA2 genes may be involved in fertility [1–4]. In fact, patients with these mutations seem to undergo a premature occult ovarian insufficiency [5, 6].

Premature ovarian insufficiency (POI) affects 1 out of 100 women over the age of 40 and 1 in 1000 woman younger than 30 years old [7].

According to the European Society of Human Reproduction and Embryology (ESHRE) the POI is characterized by the presence of amenorrhea for 4 or more months before the age of 40 and FSH level > 25 IU/l on two different measurements 4 weeks apart.

Welt et al. [8] suggested that POI represents an ovarian condition that passes through an “occult” clinical state (reduced fertility with normal FSH levels and regular menstruation) to a “biochemical” (reduced fertility, high but not menopausal FSH levels and regular menstrual cycle) until an “over” state occurs (corresponding to the POI final state).

Ovarian premature insufficiency (POI) is directly associated with diminished ovarian reserve (DOR).

DOR is defined as “A condition of reduced fecundity related to diminished ovarian function based on clinical assessment; often indicated by FSH > 10 mIU/mL or AMH < 1.0 ng/mL”, and it is associated with a reduction in the quality and quantity of oocytes.

DOR is considered a physiological condition in mid-40s women; however, it becomes pathological when it is detected in younger patients leading to POI [9].

The relatively low detectability of DOR made it difficult to draw clear pathophysiologic links between these conditions and BRCA mutations.

Furthermore, an important limitation in the evaluation of ovarian insufficiency is the difficulty in assessing their natural menopause occurrence due to their frequent choice of a bilateral salpingo-oophorectomy taken to improve the prognosis. Therefore, studies on natural menopause in mutation carriers had important limitations [10–12].

Furthermore, it has been shown that cancer occurrence itself might have an independent impact on ovarian stimulation outcome [13, 14]. Due to the high prevalence of oncologic events in BRCA1 and 2 patients, it is necessary to clarify whether these events and not BRCA mutations per se are at the basis of poor ovarian outcomes in these women. It would be of particular interest to assess which is the relevance of these oncologic events as confounding factors in BRCA1/2 women reduced fertility. [13, 15–17]

The previous six studies related to this topic, addressing ovarian stimulation in BRCA 1/2 subjects, did not analyze in detail the potential role of oncologic history itself, independent of BRCA mutations, as fertility performance determinant [16, 18–22].

The aim of this prospective study is to determine the impact of BRCA1 and BRCA2 mutations on female fertility using fertility preservation technique outcomes as primary evaluation. BRCA1 and BRCA2 breast cancer patients’ fertility were compared to non-BRCA-mutated women with breast cancer and to healthy controls.

## Materials and methods

A total of 67 breast cancer patients between 18 and 40 years old undergoing a fertility preservation treatment at the University of Bologna infertility and IVF Unit were considered. Data were collected between 1st of January 2014 and 30th of June 2019.

Inclusions criteria for the study were age between 18 and 40 years old, BMI between 18 and 28, breast cancer neoplasm stage I–II according to American Joint Committee on Cancer classification (2017) and no metastasis.

**Fig. 1:**
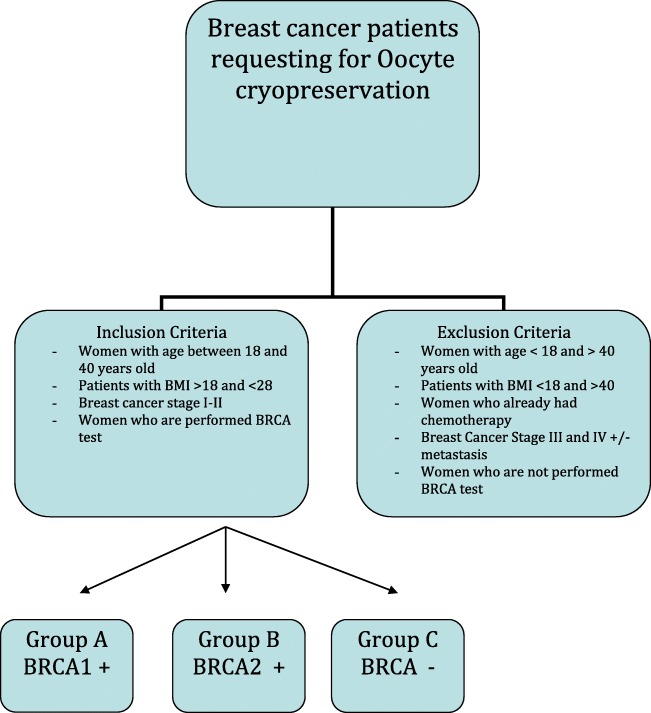
Flowchart for the study

Exclusion criteria: age over 40 years old, BMI < 18 and > 28, breast cancer neoplasm stage III–IV and do not performed the BRCA test. A total of 21 patients had not performed the test. Patients were divided into four groups: Group A was composed by 11 BRCA1-mutated breast cancer patients, Group B was composed by 11 BRCA 2 mutated breast cancer patients, Group C was composed by 24 breast cancer patients who performed the genetic test and resulted negative (Fig. 1).

Finally, those groups have been compared to Group D composed by 181 healthy women with a mean age of 32.4 ± 2.8, who underwent ovarian stimulation due to male factor.

Breast cancer patients, in groups A, B, and C, were referred to our center for fertility preservation before or after breast surgery and before chemo and radiotherapy treatments.

Among the 46 recruited patients, 26 had a hormone sensitive neoplasm evaluated with immunoistochemical analysis of excised neoplastic tissue. Five of the 46 patients had BRCA1 mutation (Group A) and 7 had BRCA 2 mutation (group B), while 14 subjects had no BRCA mutations (Group C).

AMH, AFC, and FSH were assessed in day 3 of the follicular phase prior to the stimulation to assess their basal ovarian reserve.

All patients were stimulated with r-FSH starting dose 150 IU and GnRH analog long protocol. In breast cancer patients with hormone-sensitive neoplasm, letrozole (5 mg daily) was added from the first day of the ovarian stimulation with the last administration the day before the oocytes pick up. After the egg collection, the letrozole has been continued until the estradiol blood levels reached 50 pg/mL [23].

A close monitoring was performed with seriated estradiol blood tests and pelvic ultrasounds in order to measure the follicles diameter. When the follicles reached a diameter of 18 mm and the estradiol serum levels was considered appropriate, oocyte maturation was induced with the administration of hCG 36 h prior to the transvaginal ultrasound guided ovum pickup.

Ethical approval was obtained from the local Ethical Committee.

### Statistical analysis

Data were expressed as mean ± SD. Qualitative data were analyzed using Fisher’s exact test. The significance of between-group differences was assessed using ANOVA.

BMI and age were controlled in the adjusted models. Statistical significance was reached at *p* < 0.05.

## Results

The main results are shown in Table 1.

**Table 1:** Basal values and results of ovarian stimulation in the four groups

	**Group A** (BRCA1 +) (*n* = 11)	**Group B** (BRCA2 +) (*n* = 11)	**Group C** (Breast cancer BRCA -) (*n* = 24)	**Group D** (Controls) (*n* = 181)	***p***
AGE (m ± ds) (range)	31.5 ± 3.2 (27–36)	33.2 ± 4.5 (24–40)	32.5 ± 4.3 (20–40)	32.4 ± 2.8 (26–41)	n.s.
AMH (ng/ml) (m ± ds) (range)	1.2 ± 1.1* (0.4–2.8)	4.4 ± 5.3 (0.6–6.3)	4.5 ± 4.1* (0.9–15.6)	3.8 ± 2.5* (1.1–10.5)	≤ 0.05
BASAL FSH (mUI/ml) (m ± ds) (range)	6.2 ± 3,8 (2.2–9.8)	5.9 ± 1.9 (4.2–7.5)	6.5 ± 2.7 (2.9–10.9)	7.1 ± 2.2 (3.2–9.7)	n.s.
AFC (m ± ds) (range)	11.8 ± 8.5 (1–15)	11.5 ± 4.9 (8–20)	14.2 ± 7.5 (5–35)	12.48 ± 1.4 (6–28)	n.s.
Days of stimulation (m ± ds) (range)	11.6 ± 3.6 (7–15)	10.2 ± 2.8 (7–17)	10.8 ± 2.6 (8–13)	11.3 ± 2.5 (9–14)	n.s.
Total dose of gonadotropins (U.I.) (m ± ds) (range)	2206 ± 1392* (900–4500)	2047,5± 829* (900–3375)	2106.3± 1336* (825–3300)	1597 ± 709 (1050–3050)	≤ 0.05
Developed follicles (m ± ds) (range)	12.5 ± 4.7 (5–22)	13.4 ± 5.5 (6–27)	13.1 ± 5.7 (3–24)	12.3 ± 5 (6–23)	n.s.
Retrieved oocytes (*n*) (m ± ds) (range)	6.7 ± 4.9 (2–18)	10 ± 6.8 (2–27)	9.1 ± 6.1 (1–23)	8.8 ± 4.3 (2–21)	n.s.
Cryopreserved oocytes MII (n) (m ± ds) (range)	3.1 ± 2.3* (0–14)	7.6 ± 5.9 (1–22)	7.2 ± 4.4* (1–15)	7.3 ± 3.4* (1–17)	≤ 0.05

Group A BRCA1 + cancer patients

Group B BRCA2 + cancer patients

Group C cancer patients BRCA-

Group D controls

In Group A, patients showed significant lower AMH levels compared to both Group C and D (Group A 1.2 ± 1.1 vs Group C 4.5 ± 4.1 *p* < 0,05 and Group A 1.2 ± 1.1 and Group D 3.8 ± 2.5 p < 0,05).

Comparing BRCA1 and BRCA2 patients, AMH showed a borderline difference that, however, did not reach a statistical significance (*p* = 0,069).

Moreover, BRCA1 patients showed a significantly lower rate of mature oocytes (MII) suitable for cryopreservation compared to Group C and D (3.1 ± 2.3 vs 7.6 ± 5.9 vs 7.2 ± 4.4 *p < 0,05*), while immature oocytes retrieved (MI and GV) were not cryopreserved.

No differences were found in the total number of follicles and oocytes retrieved between the four groups.

The other important aspect considered was the total dose of gonadotropins used. A higher dose of r-FSH was needed in groups A, B, and C compared to group D (Group A 2206 ± 1392.4, Group B 2047.5 ± 829.9, Group C 2106.3 ± 1336.1, Group D 1597 ± 709).

## Discussion

The present study showed that BRCA 1 patients have a higher risk of premature ovarian failure confirmed by a diminished ovarian reserve, sustained by a lower AMH (an important ovarian reserve serum marker) and a lower mature oocytes’yield.

Therefore, the hypothesis of the occult primary ovarian insufficiency is supported by this study.

On the contrary, no statistical differences detected in BRCA2-mutated patients confirm that this gene might not impact fertility in a relevant manner.

Oktay et al. [5] obtained similar results correlating BRCA1 with occult primary ovarian insufficiency. Nine BRCA1 and four BRCA2 patients underwent an ovarian stimulation in order to preserve fertility. No statistical difference in oocyte yield was found between BRCA2 and controls.

Derks-Smeets [16] considered 18 BRCA1 and 20 BRCA2 mutation carriers compared to 154 control patients. This retrospective study showed that BRCA1 mutation carriers produced lower mature oocytes; moreover, the study reported a lower but not significant pregnancy rate in these patients. According to his study, there were no differences in BRCA2 carriers and controls.

A prospective study by Turan [20] also confirmed that BRCA1 had lower oocyte yield compared to BRCA negative and untested patients, also reporting the same findings for BRCA2 mutated patients.

Shapira et al. [19] showed no differences analyzing ovarian stimulation. Forty-three BRCA1 carriers, 17 BRCA2, and 1 BRCA1 and BRCA2 carriers were compared to 62 controls; no differences were found in the number of retrieved oocytes or clinical pregnancy.

Another study published on this topic was the one by Lambertini et al. [18] which compared only breast cancer patients with and without BRCA mutations. The study showed that BRCA-positive women tend to recover fewer oocytes than BRCA-negative breast cancer patients. However, the study did not consider the possible effect of the cancer on the ovarian stimulation outcome and did not compare the results with a control group.

In the present study, ovarian response can be modified by the influence of cancer. In fact, the study shows that even breast cancer patients with no mutation needed a higher dose of gonadotropins for ovarian stimulation.

In the latest retrospective study by Guannala et al. [21], a BRCA+ and − cancer and a cancer-free cohort were analyzed. Thirty-eight BRCA+ breast cancer patients were compared to 53 BRCA- breast cancer patients and 19 BRCA+ carriers were compared to 600 women undergoing elective egg freezing. No differences were found in ovarian reserve and stimulation between the BRCA + and – women. These conflicting results address the need to enlarge the number of studies on this topic.

Many studies set AMH as their primary objective. The purpose of these studies was to trace a possible negative effect of BRCA mutation on AMH blood levels.

In 2013, a study by Titus et al. showed a statistical difference in AMH levels between BRCA1 carriers and controls (*p* < 0.0001), but the same difference was not found between BRCA2 and controls.

These results could be explained by the fact that BRCA1 undergoes a copious reduction with age. BRCA 2 instead appears to decrease in a normal range [22].

Even if some evidence showed a reduction in the AMH level even in BRCA2 mutation carrier patients, this reduction has proved to be not significant [24]. Three other studies obtained a similar result: A significant difference in AMH levels between BRCA1 carriers but no BRCA2 and control was found in Philips, Wang, and Johnson’s studies [3, 24, 25].

Michaelson Coen [26] and Van Tilborg [27] found no difference in AMH levels between BRCA mutation carriers and noncarriers.

On the other hand, Giordano et al. [28] investigate exclusively BRCA1 and found a significantly lower AMH level than controls.

Very few of the previously published studies analyzed the differences between the retrieved oocytes and the oocytes suitable for cryopreservation (MII). The results of this study highlight the hypothesis that BRCA1 plays an important role in repairing oocytes damage. The direct proof of this hypothesis is still lacking, but recent clinical and laboratory studies suggest that the damage tends to accumulate more often on these women’s oocytes and that these damages are less prone to be repaired due to the lack of the main repair gene of oocytes [29].

Consequentially, it can be hypothesized that the effect of the missing BRCA1 repair proteins prevents the damaged oocytes from proceeding in the metaphase II of the meiotic division, a necessary step for the maturation and the subsequent fertilization [30].

This could explain the reported differences among mature oocytes retrieval in mutation carriers.

However, not many previous studies have addressed the relevant issue of a potential interference of independent cancer-related determinants of infertility on the results obtained in BRCA1-mutated women. Many of these patients, indeed, may have had a cancer history (due to the intrinsic characteristics of BRCA mutation to dramatically increase cancer prevalence). Such a high prevalence of an oncologic history may represent a relevant confounding factor in the interpretation of the role of BRCA1 mutation in the result of ovarian stimulation.

In order to overcome this bias, BRCA-mutated women with breast cancer were compared to both other breast cancer patients with negative genetic test and with a control group. To ensure the reliability of the comparison, the control group was composed of women undergoing an ovarian stimulation due to male factor.

The results of our study suggest that BRCA1 (but not BRCA2)-mutated women with no history of cancer have an independent reduction in the response to ovarian stimulation. The analysis of the ovarian profile in this subgroup of BRCA1 and 2 as well as cancer patients with no mutation showed the need for a greater amount of gonadotropins to obtain effective stimulation.

This evidence has its relevance in the fact that continued progress in the diagnosis and treatment of malignant tumors has led to significant improvements in cancer survival. Nowadays, 95% of early-stage breast cancer patients with local disease and 84% with regionally confined disease survive [31].

More aggressive screening and treatment innovation heightened public awareness leading to higher survival rates that are destined to increase even more, allowing more patients to enjoy a near normal life after cancer. Infertility and/or sterility often occur alongside cancer cure and are known to negatively impact posttreatment quality of life. Goodwin et al. [32] estimated that 53% to 89% of premenopausal breast cancer patients treated with alkylating chemotherapy experienced premature menopause, and the risk was strongly associated with higher age at the time of treatment. Oocytes cryopreservation represents a powerful strategy for fertility preservation in these patients [33–35].

Therefore, the storage of oocyte can be a concrete, pragmatic tool to preserve fertility in oncological patients. Furthermore, egg freezing can evade ethical and legal concerns unlike embryo cryopreservation, despite this is believed to be the gold standard for fertility preservation.

This study certainly has some limitations, mainly the small number of BRCA1 and BRCA2 carriers included, and it highlights the need of a prospective multicentric study to overcome these limitations.

Moreover, the study highlights the need for a proper counseling to oncological women regarding their risk of a premature ovarian insufficiency induced by chemotherapy.

## Conclusions

In conclusion, this study suggests that BRCA1 mutation carriers have a significantly lower mature oocytes yield. This effect appears to be independent of the potential interference of cancer. Such an independence of BRCA1 mutation role was confirmed by the comparison of this study group with the group of patients without BRCA mutations treated for breast cancer.

Further studies on the impact of BRCA genes on fertility are needed to confirm our findings and provide a better understanding of the underlying pathophysiology.

Oocytes cryopreservation is a feasible option that should be suggested to these patients, especially considering the frequent recommendation of a prophylactic bilateral salpingo-oophorectomy at the age of 40 for both BRCA1 and 2 women [36].
